# Germanium silicon oxide achieves multi-coloured ultra-long phosphorescence and delayed fluorescence at high temperature

**DOI:** 10.1038/s41467-022-32133-2

**Published:** 2022-08-01

**Authors:** Huai Chen, Mingyang Wei, Yantao He, Jehad Abed, Sam Teale, Edward H. Sargent, Zhenyu Yang

**Affiliations:** 1 MOE Laboratory of Bioinorganic and Synthetic Chemistry, Lehn Institute of Functional Materials, School of Chemistry, Sun Yat-sen University, Guangzhou, 510275 Guangdong China; 2 Department of Electrical and Computer Engineering, University of Toronto, 10 King’s College Road, Toronto, M5S 3G4 Ontario Canada

**Keywords:** Lasers, LEDs and light sources, Materials chemistry, Materials for optics

## Abstract

Colour-tuned phosphors are promising for advanced security applications such as multi-modal anti-counterfeiting and data encryption. The practical adoption of colour-tuned phosphors requires these materials to be responsive to multiple stimuli (e.g., excitation wavelength, excitation waveform, and temperature) and exhibit excellent materials stability simultaneously. Here we report germanium silicon oxide (GSO) – a heavy-metal-free inorganic phosphor – that exhibits colour-tuned ultra-long phosphorescence and delayed fluorescence across a broad temperature range (300 – 500 K) in air. We developed a sol-gel processing strategy to prepare amorphous oxides containing homogeneously dispersed Si and Ge atoms. The co-existence of Ge and Si luminescent centres (LC) leads to an excitation-dependent luminescence change across the UV-to-visible region. GSO exhibits Si LC-related ultra-long phosphorescence at room-temperature and thermally activated delayed fluorescence at temperatures as high as 573 K. This long-lived PL is sensitized via the energy transfer from Ge defects to Si LCs, which provides PL lifetime tunability for GSO phosphors. The oxide scaffold of GSO offers 500-day materials stability in air; and 1-week stability in strong acidic and basic solutions. Using GSO/polymer hybrids, we demonstrated colour-tuned security tags whose emission wavelength and lifetime can be controlled via the excitation wavelength, and temperature, indicating promise in security applications.

## Abstract (web summary)

Advanced security applications require materials responsive to different stimuli with remarkable stability. Here, Sargent et al. introduce Ge homogenously into a silica scaffold and obtain a colourtuned germanium silicon oxide with ultra-long phosphorescence and delayed fluorescence across a broad temperature range.

## Introduction

Colour-tuned phosphors are luminescent materials whose photoluminescence (PL) properties (emission colour, intensity, and PL lifetime) are responsive to external stimuli such as excitation wavelength, waveform, power intensity, and temperature^1–4^. Colour-tuned phosphors have potential applications in high security-level anti-counterfeiting and data storage/encryption^5–7^. The anti-counterfeiting marks made with colour-tuned phosphors show variable optical responses under external stimuli, therefore less likely to be copied using conventional security phosphors^8,9^. Tunable PL properties of colour-tuned phosphors provide multi-dimensional optical codes to store sensitive information, an approach that offers the potential to improve encoding capacity in optical data encryption^10^.

Recently, organic colour-tuned phosphors have attracted interest in view of their versatility in molecular design and their non-reliance on toxic metal ion activators^1,6,11^. This has brought new application opportunities in security applications. However, the practical application of organic colour-tuned phosphors is impeded by their lack of long-lasting durability under a range of relevant environmental conditions (Supplementary Table 1), since their organic backbones exhibit limited resistance to heat, humidity, and oxidation^12–16^.

Inorganic colour-tuned phosphors are well-suited to practical application in light of their high materials stability. Inorganic phosphors—including heavy-metal-doped inorganic phosphors and colloidal quantum dots—have been engineered to display colour-tuned fluorescence and phosphorescence^17–21^. Their luminescent properties are controlled by excitation wavelength, power intensity, and temperature^22–25^. Until now, though, inorganic colour-tuned phosphors have yet to report stability against moisture and thermally induced degradation^26,27^.

Silica (SiO_2_) provides robust silicon-oxygen bonds and thus is well suited to serve as a protective host for both inorganic and organic phosphors^21,28^. Employing a rigid silica scaffold overcomes the thermal quenching of excitons, stabilizing photoluminescence at high temperatures^21^. Silica has also been considered an attractive phosphor in light of its Si-defect-related luminescence^29^. However, there has been no demonstration of colour-tuned luminescence by engineering the Si defects for silica phosphors, nor has the expected thermal stability been thoroughly investigated. This we assign to limited control over defect formation in silica phosphors^30^.

Here we incorporated Ge into an amorphous silica scaffold to produce a material containing multiple luminescent centres (LCs) which allowed us to unite stability with colour-tuned luminescence^31–34^. We developed a sol-gel-based materials processing strategy to introduce, in an anatomically homogeneous fashion, Ge atoms to form germanium silicon oxides (GSO). By implementing the selective excitation of LCs, we tuned the emission colour of GSO across the blue-to-yellow visible light region. We found energy transfer between Ge and Si defect states stabilizes the phosphorescence lifetime of GSO to a long 0.6 s at room temperature. We observed thermally activated delayed fluorescence (TADF) at high temperatures (300–500 K) in GSO, leading to temperature-dependent colour tuning. The luminescence features of GSO have no observable changes the following storage under ambient conditions for over 500 days, and they exhibit good stability against acidic or base solutions. We fabricated multi-mode anti-counterfeiting patterns using GSO and demonstrated tri-modal optical encryption by combining GSO with pristine silica phosphors. The results indicate the potential of GSO as a stable, heavy-metal-free security phosphor with multi-dimensional colour tunability.

## Results and discussion

### Excitation-wavelength-dependent colour tuning in GSO

The excitation wavelength is commonly used to tune the photoluminescence (PL) properties of colour-tuned phosphors^1,6,9,12^. Typically, colour-tuned phosphors contain multiple LCs with distinct excitation bands^24^. Excitation-wavelength can therefore selectively control the excitation of LCs, changing the emission colour and PL lifetime accordingly.

**Fig. 1:**
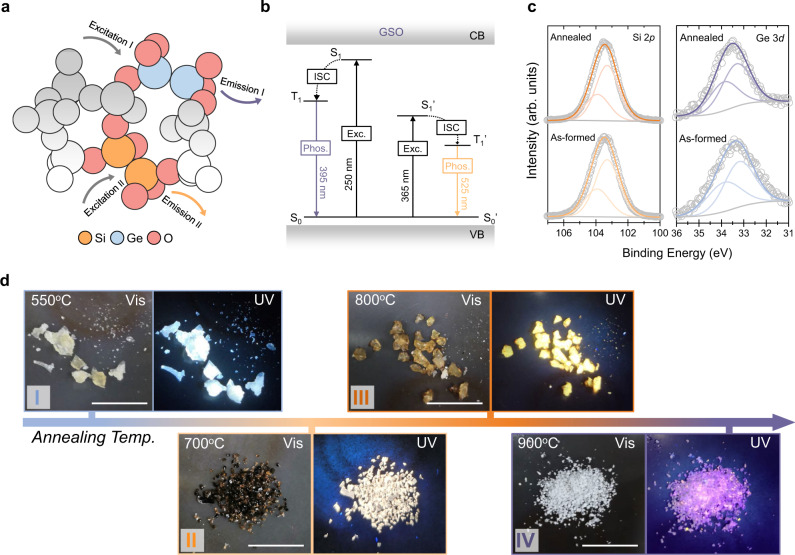
Design and synthesis of GSO phosphors. **a** Rational design of multicomponent GSO for colour-tuned phosphorescence. The faded molecules refer to the amorphous SiO_2_ scaffold. By using different excitation wavelengths (excitation I or excitation II), one may selectively excite Ge- and Si-based LCs, demonstrated by different emission wavelengths (emission I or emission II). **b** Diagram of the proposed energy levels of Ge oxygen-deficient centre (GeODC(II)) and Si LCs which coexist in GSO solids. VB valence band, CB conduction band, Exc. excitation, ISC intersystem crossing, S_1_ the lowest excited singlet state, and T_1_ the lowest triplet state. **c** High-resolution XPS results of Sample III showing silicon 2*p* and germanium 3*d* signals. Fitting results are shown for Si (2*p*_1/2_, 2*p*_3/2_) and Ge (3*d*_3/2_, 3*d*_5/2_) components. **d** Images and photoluminescence (PL) spectra of GSO powders after 1 h of annealing at various temperatures (I: 550 °C; II: 700 °C; III: 800 °C; IV: 900 °C). Excitation wavelength: 365 nm for Samples I, II, and III; 254 nm for Sample IV. Scale bar = 1 cm.

We sought to design oxide phosphors with multiple, distinct LCs. We reasoned that pristine silica phosphors are unlikely to achieve this goal: Si LCs are generated under specific synthesis conditions (including temperature, atmosphere, and precursors), and this limits the coexistence of multiple Si LCs^21^. Ge oxygen-deficient centres (GeODC(II)) can be easily formed under various conditions in germanosilicate^35^. When GeODC(II) and Si LCs with distinguishable PL properties coexist compatibly within GSO, the emission of GSO can potentially be tuned using different excitation wavelengths (Fig. 1a, b). This constitutes the basis for colour-tuned security applications.

Here, GSO was synthesized by homogeneously mixing tetramethoxygermanium (TEOG) and tetramethoxysilane (TMOS) in the sol-gel precursors and subsequent thermal annealing under an Ar/H_2_ atmosphere. Inductively coupled plasma-atomic emission spectrometry (ICP-AES) shows that the Ge content in GSO is 5.2 ± 0.2 wt%. Energy-dispersive X-ray spectroscopy (EDS) mapping further shows that the Ge element was distributed uniformly within GSO powders (Supplementary Fig. 1). The crystallinity and local elemental environment of the as-synthesized GSO were investigated using powder X-ray diffraction (PXRD), X-ray photoelectron spectroscopy (XPS), and X-ray absorption spectroscopy (XAS). No crystalline feature is seen in PXRD either before or after annealing, indicating an amorphous crystalline structure (Supplementary Fig. 2). From XPS, we rule out the formation of silicon or germanium nanoparticles since no signal from elemental silicon or germanium (i.e., Si^0^ and Ge^0^) is observed (Fig. 1c). Scanning electron microscope (SEM) image of GSO samples further confirm that they are bulk powders (Supplementary Fig. 1). In the XAS spectra, characteristic SiO_2_- and GeO_2_-like signals are already present in the Si K-edge and Ge L-edge, respectively for the sol-gel precursors, while thermal annealing induces negligible local environment changes to Si and Ge atoms (Supplementary Fig. 3). Taking these results in combination, we conclude that the GSO is an amorphous germanosilicate, with Ge and Si LCs homogenously mixed even following thermal annealing.

**Fig. 2:**
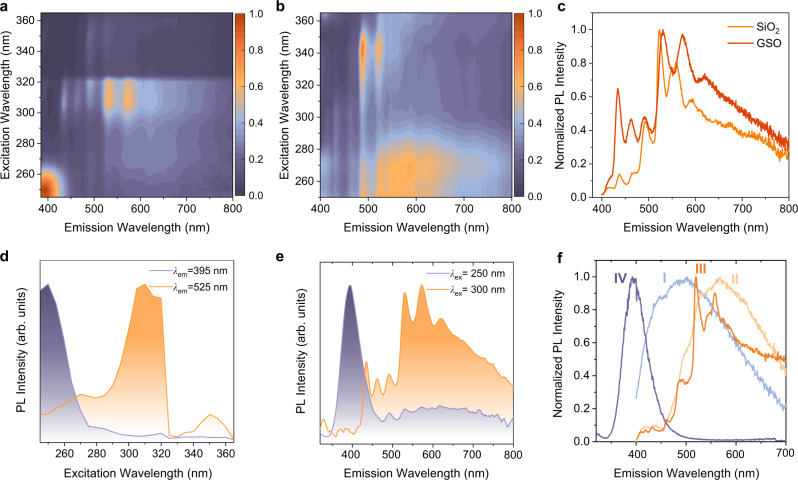
Excitation-wavelength-dependent colour tuning in GSO. Excitation-emission matrix **(**EEM) spectra of (**a**), Sample III and (**b**), pristine silica measured at room temperature under ambient conditions. Colour bars show normalized PL intensity. **c** Normalized PL spectra of pristine silica and Sample III under the same annealing procedure. Excitation wavelength = 300 nm. **d** PL excitation (PLE) spectra and (**e**) photoluminescence (PL) spectra of GSO. *λ*_em_ emission wavelength. *λ*_ex_ excitation wavelength. **f** PL spectra of GSO powders after thermal annealing at various temperatures (I: 550 °C; II: 700 °C; III: 800 °C; IV: 900 °C) (excitation wavelength: 365 nm for Samples I, II, and III; 254 nm for Sample IV).

To investigate the colour tunability of GSO, excitation-emission matrix (EEM) measurements were carried out for both GSO and pristine silica (Figs. 2a, b). Two PL species are identified in the EEM spectra of GSO: a narrowband PL centred at 395 nm and a broadband PL ranging from 400 nm and 800 nm. The narrowband PL—absent from the EEM spectra of undoped silica—corresponds to an excitation around 250 nm with a PL lifetime of 110 μs, which are phosphorescence characteristics of GeODC(II)^36^ (Fig. 2b, Supplementary Fig. 4). The broadband PL is observed both in GSO and undoped silica with consistent PL features (Fig. 2c). We attribute the narrowband PL to GeODC(II) and broadband PL to Si-related LCs, respectively. This evidence shows that GSO contains multiple LCs, which enables excitation wavelength-dependent colour tuning.

Colour-tuned PL is demonstrated in GSO through the selective excitation of different LCs: since the excitation spectra of narrowband PL and broadband PL exhibit small overlap, the violet PL from GeODC(II) dominates the PL spectra for the excitation wavelengths from 220 to 280 nm (Fig. 2d); In comparison, the white-coloured PL from Si LCs becomes prominent in the PL spectra resulting from an excitation wavelength of around 300 nm and 360 nm (Fig. 2e). The PL peak wavelength is thereby shifted by about 130 nm (from 395 nm to 525 nm) for GSO using excitation wavelength tuning. This apparent emission colour change is favourable for security applications, as it allows the reliable readout of encrypted information by the naked eyes^37^.

Varying annealing temperatures (550–900 °C) during synthesis enabled us to further engineer silica LCs in GSOs (Fig. 1d): PL can be shifted for annealing temperatures from 550^o^C to 900^o^C under UV illumination (Fig. 2f and Supplementary Fig. 5), while diminished for an annealing temperature above 900 °C. The engineered local structure of silica LCs was directly evidenced in the electron paramagnetic resonance (EPR) spectra (Supplementary Fig. 6, Supplementary Table 2, and Supplementary Note 1), while PXRD and Fourier transform infrared spectroscopy (FTIR) analyses reveal that GSOs preserved the amorphous crystalline structure at different annealing temperatures (Supplementary Fig. 7). In addition, we observed a PL wavelength shift for this emission band following Ge incorporation. By tracking the PL spectra of GSOs as a function of Ge concentration (Supplementary Fig. 8), we found this wavelength shift is continuous, a finding we attribute to the local structure change in LCs upon the incorporation of Ge (Supplementary Fig. 9).

### PL lifetime-tuning via the introduction of Ge

**Fig. 3:**
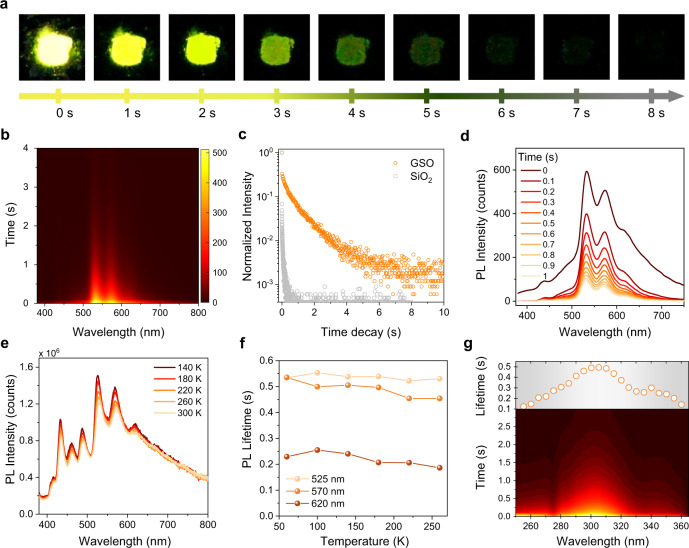
Stabilization of ultra-long phosphorescence by the incorporation of Si and Ge centres. **a** time-scale photographs of GSO powder recorded after the 365 nm UV excitation was switched off. **b** Time-resolved emission spectroscopy (TRES) of Sample III recorded at 300 K after the removal of the 300 nm excitation. The pseudo-colour change from bright yellow to dark red indicates the gradual decrease of emission intensity after removing the excitation source. The colour bar shows PL counts. **c** PL lifetime decay curves of sample III (GSO annealed at 800^o^C) and control sample SiO_2_, *λ*_ex_ = 300 nm. *λ*_em_ = 525 nm. **d** Time-resolved PL spectra of Sample III recorded at 300 K after removing the 300 nm excitation. Peak positions are unchanged throughout the long PL lifetime and are consistent with steady-state PL. **e** Temperature-dependent steady-state PL spectra of Sample III recorded under 300 nm excitation for a temperature range of 140–300 K. **f** Temperature-dependence of PL lifetimes for each of the long-lived decay components (525 nm, 570 nm, and 620 nm) recorded from 60 to 260 K. **f** EEM spectra of Sample III measured at room temperature under ambient conditions. **g** Time-resolved excitation spectra of GSO obtained at 300 K by monitoring the emission feature at 525 nm.

We found the presence of Ge stabilizes the otherwise short-lived phosphorescence from Si LCs. This enables PL lifetime-tuning in GSO and pristine silica: After switching off a 365 nm light illumination, yellow-coloured afterglow can be observed by the naked eye from 800^o^C-annealed GSO (Fig. 3a, Supplementary Movie 1), while no detectable afterglow is observed from pristine silica and GSOs annealed at other temperatures.

The afterglow properties of GSO were studied using time-resolved emission spectroscopy (TRES). As seen in Fig. 3b, a slow decay in intensity is apparent after removing the excitation source. We fit the slow decay curves with a biexponential function and obtained a PL lifetime of 0.58 s for the 525 nm emission, which is comparable with that of ultra-long organic phosphorescence^7,12,13^(UOP, Fig. 3c). Comparing the time-resolved PL profiles (Fig. 3d) and steady-state PL spectra (Fig. 2c), we further confirm that this yellow (peaks at 525, 570 and 620 nm) long-lived emission is stabilized ultra-long phosphorescence (UP) from Si LCs.

To examine the mechanism of UP stabilization for GSO, we carried out temperature-dependent PL measurements from 140 K to 300 K. The slight thermal quenching resulted in a minor decrease in intensity in temperature-dependent PL spectra (Fig. 3e) and negligible change in PL lifetime (Fig. 3f and Supplementary Fig. 10). Therefore, we rule out thermal-activated electron de-trapping—usually observed in Ce^3+^-doped inorganic phosphors—as the mechanism of UP stabilization for GSO^25^.

Time-resolved excitation spectra of GSO reveal that UP is generated upon excitation at 310 nm (Fig. 3g). We found that this coincides with one of the excitation bands of Si LCs as seen in EEM spectra (Fig. 2a). This excitation band—negligible in the EEM spectra of undoped silica (Fig. 2b)—is introduced upon Ge incorporation. The intrinsic excitation band of Si LCs does not generate UP and exhibits weaker excitation intensity than does the 310 nm band in GSO (Fig. 2a). From these findings, we offer that Ge incorporation introduces new defect states that sensitize Si LCs, while pristine silica does not emit long-lived PL because of the lack of Si LC sensitization (Fig. 3b). The mechanism we present here is akin to the sensitizer-activator co-doping effect in lanthanide-doped inorganic phosphors^38^.

To investigate this mechanism of UP stabilization, we carried out EEM measurements on GSOs with varied Ge concentrations (Supplementary Fig. 11). We found that the 310-nm extrinsic excitation band was observed only with a Ge concentration of 1:20. This corresponds to the coexistence of Si- and Ge-related paramagnetic defects exclusively at the same doping level (Supplementary Fig. 12, Supplementary Table 3). We conclude that the extrinsic excitation band is related to energy transfer among defects. When we vary the annealing atmosphere of GSOs, we find evidence that Si- and Ge-related paramagnetic defects are H(I) and H(II) centres, respectively, since they are created only under a mixed H_2_/Ar atmosphere (Supplementary Fig. 13, 14)^39^. Detailed discussions about the EPR results are available in Supplementary Note 1.

UP from GSO exhibits excellent stability against oxygen- and moisture-induced degradation: no obvious phosphorescence lifetime changed after over 500 days of storage in air (Supplementary Fig. 15) and after over 7 days of immersion in strongly acidic and basic solutions (Supplementary Fig. 16). These significantly exceed the stability level of UOP and are ideal for security printing on banknotes and trademarks, which require air and water stability^37^.

### Temperature-dependent colour tuning in GSO

Thermal quenching limits the phosphorescence lifetime of UOP at high temperatures. However, GSO may overcome this issue, as the rigid oxide scaffold is expected to reduce thermally induced nonradiative recombination^27,28^. Temperature can also act as an external stimulus for colour-tuned phosphors^40^. We sought therefore to study the PL properties of GSO at elevated temperatures.

**Fig. 4:**
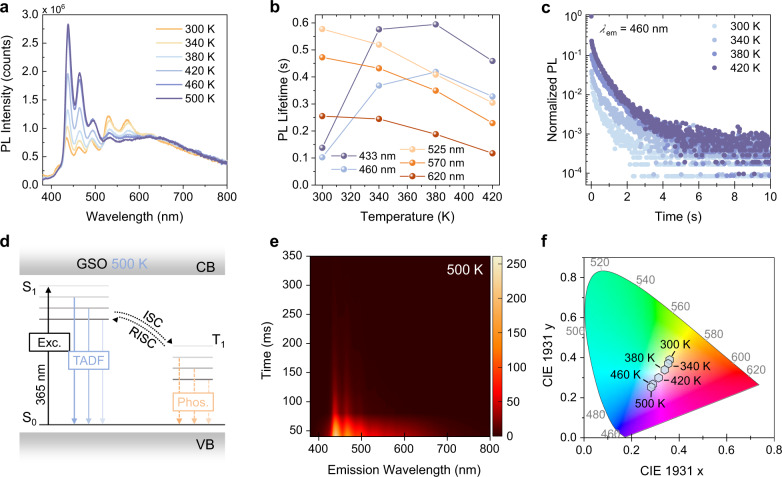
Photophysical properties of GSO at elevated temperatures. **a** Temperature-dependent PL spectra (휆_ex_ = 300 nm). **b** Temperature-dependent PL lifetimes at the major emission peaks. **c** Normalized temperature-dependent PL decay curves from 300 to 420 K (*λ*_em_ = 460 nm) **d** Proposed energy transfer processes of Sample III at high temperatures. Blue-band TADF is generated through the reverse intersystem crossing (RISC) from the lowest excited triplet state (T_1_) of the silica LCs. **e** Time-resolved emission spectroscopy (TRES) recorded at 500 K using 300 nm excitation. **f** Temperature-dependent CIE chromaticity diagram for the PL of Sample III from 300 to 500 K.

We noticed that the fluorescent blue-band PL (i.e., 400-500 nm) is thermally activated at high temperatures (300–500 K), as evidenced by the enhanced PL intensity, the prolonged PL lifetime, and the increased ratio of the long component decay in the total PL decay (Fig. 4a–c). Notably, the PL lifetime at 433 nm can reach a maximum of 0.59 s at a high temperature of 380 K, similar to the longest phosphorescence lifetime (0.58 s) at room temperature (Fig. 4b). Combining these results, we reason that the blue-band afterglow at high temperatures is derived from thermally activated delayed fluorescence (TADF)^41,42^. This is generated via reverse intersystem crossing (RISC) from triplet states to singlet states at the silica LCs, with the aid of thermal energy (Fig. 4d).

Thermally induced PL quenching is instead observed (viz. phosphorescent yellow-band PL, i.e., PL at 500–600 nm): both the PL intensity and phosphorescence lifetime are decreased at elevated temperatures (Fig. 4a, b). Despite the existence of thermal quenching, the phosphorescence lifetime at 525 nm is still up to 0.30 s at temperatures as high as 420 K, at which UOP is diminished in most organic phosphors^12^ (Fig. 4b). This indicates the effectiveness of UP stabilization by the rigid silica scaffold.

As a result of the relative intensity change between fluorescence and phosphorescence at elevated temperatures, the emission colour of GSO shifts progressively from (0.360, 0.389) at 300 K to (0.283, 0.253) at 500 K in the CIE 1931 chromaticity diagram (Fig. 4e, f). Therefore, the temperature can be used as an external stimulus for colour-tuning GSO.

### Multimodal anti-counterfeiting and information encryption

As GSO combines excitation wavelength-dependent colour tuning, PL lifetime-tuning, and temperature-dependent colour tuning simultaneously, it is an ideal security phosphor for multimodal anti-counterfeiting and information encryption.

**Fig. 5:**
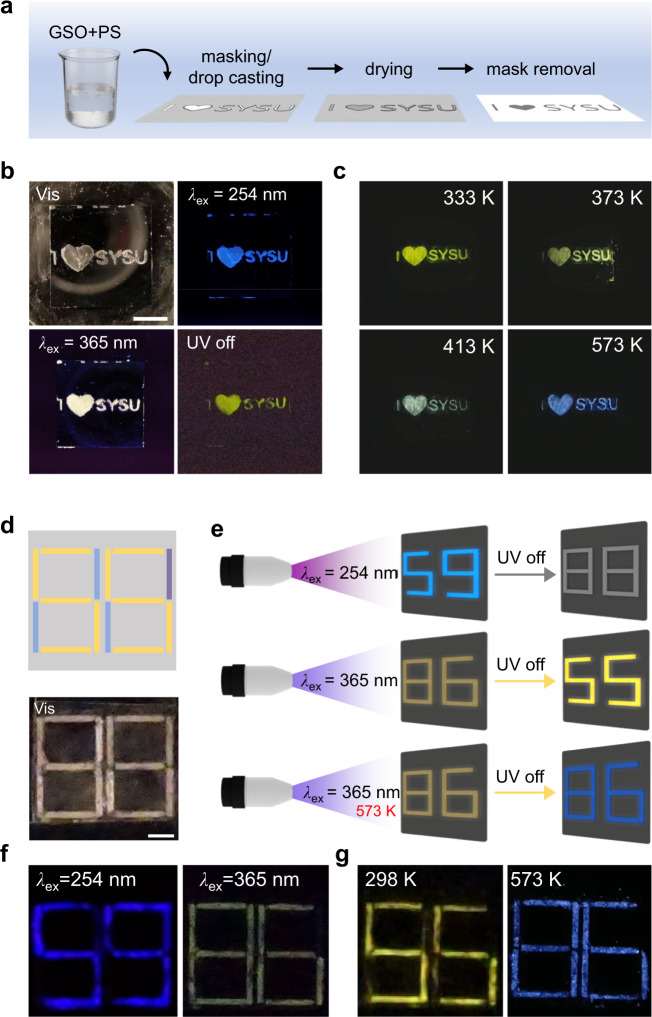
Security applications of colour-tuned GSO. **a** GSO patterning process by polymer encapsulation. **b** Anti-counterfeiting label demonstration with excitation-wavelength-dependent security features. PL wavelength and lifetime from the label are different using 254 nm and 365 nm excitation, respectively. (Scale bar = 1 cm). **c** Temperature-responsive security demonstration. The afterglow colour of the anti-counterfeiting label is tuned by varying the temperature from 333 K to 573 K. Photographs were all taken 1 s after the excitation (*λ*_ex_ = 365 nm) was switched off. **d** Schematic of the encryption pattern design. Different oxide-based phosphors were selectively deposited on the pre-designed pattern (blue: pristine SiO_2_; yellow: Sample III; violet: Sample IV, scale bar = 0.5 cm). **e** Triple-mode decoding process on colour-tuned GSO. By tuning the excitation wavelength and temperature, the pattern can display different digital numbers. **f** Demonstration of excitation-wavelength-responsive encoding. The numbers “59” and “86” are displayed under 254 and 365 nm, respectively. **g** Demonstration of temperature-responsive encoding. The yellow-coloured number “55” and the blue-coloured number “86” are displayed at 298 and 573 K, respectively, after removing the 365 nm illumination.

We fabricated anti-counterfeiting marks by drop-casting GSO/polystyrene (PS) hybrid ink on a patterned silicon wafer (Fig. 5a). Tri-modal anti-counterfeiting was demonstrated for GSO/PS-based security printing (Fig. 5b, c): When switching the excitation wavelength from 254 nm to 365 nm, the emission colour of the anti-counterfeiting patterns is changed from blue to white, as a result of the excitation wavelength-dependent colour tuning in GSO; When turning off the 365 nm UV light illumination, the yellow coloured afterglow emerged due to the stabilization of UP (Fig. 5b); When increasing the temperature from 333 K to 573 K, the emission colour of the afterglow shifted from yellow to blue progressively due to TADF (Fig. 5c).

We further demonstrated multi-modal information encryption by combining GSO and pristine SiO_2_ phosphors. As seen in Fig. 5d, a digital number pattern was prepared by arranging GSO and pristine phosphors at different locations. Two types of GSO were employed: 800 ^o^C-annealed GSO (Sample III) which exhibits excitation wavelength-dependent colour tunability, and 900^o^C-annealed GSO (Sample II) which only emits PL from GeODC(II). Using this pattern, we achieved tri-modal data encryption in response to excitation wavelength, excitation on/off, and temperature, respectively (Fig. 5e). Under the mode of excitation wavelength-encoding, the number “59” is displayed using 254 nm illumination (PL from the Sample I and II), while the number “86” is displayed under 365 nm illumination (PL from the Sample I and pristine silica) (Fig. 5f). Under PL lifetime-encoding, the number “86” is changed to “55” after switching off the excitation source since only the sample I exhibits UP. However, as both Sample I and pristine silica exhibit TADF, when excitation is removed at 573 K the number “86” is visible rather than the number “55” observed at 300 K under the temperature-encoding mode (Fig. 5g). This demonstration confirms the encoding capacity of GSO in combination with pristine silica by harnessing multi-dimensional colour tunability.

It is noted the high temperature and short excitation wavelength used to reveal the sensitive information may also cause potential safety concerns in the practical application^43^. Since GSO exhibits temperature-dependent colour tunability from 300 K to 500 K, one can select actual operating temperatures in such a way as to avoid fire hazards. Users should also be aware of health risks associated with the short excitation wavelength, and the use of an enclosure for illumination should be evaluated with this in mind.

This work demonstrates GSO as a new inorganic colour-tuned phosphor with ultra-long phosphorescence and delayed fluorescence over a broad temperature range. The colour-tuned emission across the UV and visible light region was achieved by the selective excitation of Ge and Si luminescent centres. Energy transfer between Ge and Si defects stabilized the ultra-long phosphorescence up to 0.58 s at room temperature. Thermally activated delayed fluorescence was further demonstrated in GSO at high temperatures (300–500 K). GSO exhibited over 500-day air-storage stability, 7-day water stability in strong acid/base solution, and robust thermal stability at temperatures up to 573 K. With GSO-based security inks, we produced high security-level anti-counterfeiting tags that exhibit a tri-modal optical response. We also demonstrated tri-modal data encryption using GSO and pristine silica phosphors. These new findings indicate the great potential of GSO as stable and non-toxic colour-tuned phosphors for advanced security applications.

## Method

### Materials

All chemicals used are commercially available and were used without any additional purification steps: tetramethoxysilane (TMOS, 98%), tetraethoxysilane (TEOS, 98%), nitric acid (36%), sodium hydroxide (98%), tetrahydrofuran (AR), styrene (98%) and anthracene-9,10-diyl-bis-methylmalonate (ADMA, 95%) were purchased from Aladdin Chemical Inc.; tetramethoxygermanium (TEOG, 98%) was purchased from Gelest Inc.

### Solid-state synthesis of GSO and the control sample SiO_2_

9.3 g of TMOS (60 mmol) and 0.76 g of TEOG (3.0 mmol) were added into a 100-mL Schlenk flask equipped with a magnetic stirring bar. 10 mL of 90 mM HNO_3_ and 10 mL of methanol were added to the flask with mechanical stirring at room temperature under constant nitrogen flow to initiate the sol-gel reaction. The solution gradually became white and cloudy within 5 min, and the reaction was maintained at room temperature for 24 h. After that, the white, gel-like product was isolated from the solution by vacuum filtration and subsequently dried under vacuum at room temperature for 16 h. After the drying process, the powder-like product was transferred to a 20-mL glass vial and stored in ambient conditions for further use.

2 g of the powders were then placed in a quartz reaction boat and transferred to a high-temperature tube furnace (Lindberg). The sample was heated from ambient to the pre-designed peak temperatures (550, 700, 800, and 900 °C) at 18^o^C/min in a slightly reducing atmosphere (5% H_2_ + 95% Ar). The sample was maintained at the processing temperature for 5 h and then naturally cooled down to room temperature. The resulting amber solid was ground using mortar and pestle and subsequently ground mechanically using a ball milling grinder (MSK-SFM-LN-192, MTI KJ Co., Ltd. frequency = 50 Hz, operating time = 3 h) to yield a fine greyish GSO powder. The control sample SiO_2_ was prepared following the same procedure as GSO except for the addition of TEOG. All of the products were transferred to 20-mL glass vials for storage in an ambient environment.

### Photoluminescence (PL) measurements

The photoluminescence excitation spectra (PLE) and temperature-dependent PL spectra of the GSO powdery samples were recorded using an FLS 980 spectrometer (Edinburgh Instruments) equipped with an alternating temperature module. A 450 W Xe lamp served as a continuous-wave light source in steady-state PL measurements. The excitation-emission matrix (EEM) PL spectra of GSO were recorded by a Horiba Duetta fluorometer. The temperature-dependent PL lifetime profiles and Time-resolved emission spectroscopy (TRES) of the samples were recorded by Edinburgh FLS 920 spectrophotometer equipped with a 150 W nF900 flash lamp.

### Powder x-ray diffraction (PXRD) and x-ray photoelectron spectroscopy (XPS) measurements

PXRD patterns were collected with a Rigaku Smart Lab diffractometer (Bragg-Brentano geometry, Cu Kα1 radiation, *λ* = 1.54056 Å). The spectra were generated from scans between 2θ ranges of 10°–80° with the integration of 600 s. XPS results were obtained using a VG Scientific ESCALAB 250 instrument, Thermo Fisher Scientific. CasaXPS software (VAMAS) was used to interpret high-resolution results. All spectra were internally calibrated to the C 1s emission (284.8 eV).

### X-ray absorption near-edge structure (XANES) analysis

The XAS Ge L-edge and Si K-edge spectra were measured using SGM (11ID-1) beamline at the Canadian Light Source (CLS). The samples were prepared by spreading a thin layer of powder on a piece of conductive carbon tape which was placed on a metallic holder. All measurements were taken at room temperature in the fluorescence mode using four Amptek silicon drift detectors (SDDs) simultaneously. The XAS for each sample was measured 10 times at different spots on the sample (0.1 mm separation) and then averaged. Data post-processing and fitting were done entirely using the Demeter software package^44^.

### Electron paramagnetic resonance (EPR) measurements

All EPR data was processed by continuous-wave (X-band EPR measurements on powdery samples (50 mg) which were recorded with a Bruker E500 spectrometer using a Bruker super-high Q cavity. The measurements were carried out at room temperature with a modulation frequency of 100 kHz, modulation amplitude of 0.01 mT, and a microwave power of 0.2 mW (30 dB) under non-saturating conditions.

### Anti-counterfeiting pattern preparation and tests

The pattern was pre-designed and made on plastic tape. The pattern was then transferred onto a piece of silicon wafer (3 cm * 3 cm, Guangzhou Lige technology co. LTD), and the rest of the tape was removed. 500 mg of ground GSO powder was dispersed in 10 mL of oligomeric styrene by mechanical stirring in the air at room temperature for 30 min, yielding a highly viscous suspension. The resulting suspension was subsequently drop-cast onto the pattern/silicon wafer and then dried in air at room temperature for 6 h. To test the luminescence behaviour, the film was irradiated by a 365 nm ultraviolet light source (5 W) in the air at various temperatures. For the demonstration of temperature-responsive encoding, the samples were placed on a hot plate with various temperatures which were calibrated using an infrared thermometer. (Caution: the usage of UV light could possibly cause biological damage. The high-temperature annealing and the UV-irradiation processes should be operated in the fume hood with proper personal protective equipment).

## Supplementary information

Supplementary Information

Description of Additional Supplementary Files

Supplementary Movie 1

## Data Availability

The data that support the findings of this study are available in the following repository: 10.6084/m9.figshare.19859869.v1.
