# Supplementary material for: Germanium silicon oxide achieves multi-coloured ultra-long phosphorescence and delayed fluorescence at high temperature
Source: Nat Commun. 2022 Aug 1;13:4438. doi: 10.1038/s41467-022-32133-2 (PMC9343423; doi:10.1038/s41467-022-32133-2)
Supplement: Supplementary file 1 — Supplementary Information [file 41467_2022_32133_MOESM1_ESM.pdf]

Supplementary Information for

Germanium silicon oxide achieves multi-coloured ultra-long phosphorescence and delayed-fluorescence at high temperature

Huai Chen[1†], Mingyang Wei[2†], Yantao He[1], Jehad Abed[2], Sam Teale[2], Edward H. Sargent*[2] and Zhenyu Yang*[1]

1. MOE Laboratory of Bioinorganic and Synthetic Chemistry, Lehn Institute of Functional Materials, School of Chemistry, Sun Yat-sen University, Guangzhou, 510275, Guangdong, China
2. Department of Electrical and Computer Engineering, University of Toronto, 10 King's College Road. Toronto, Ontario, Canada, M5S 3G4

†These authors contributed equally to this work.

*Corresponding authors. E-mail: ted.sargent@utoronto.ca; yangzhy63@mail.sysu.edu.cn

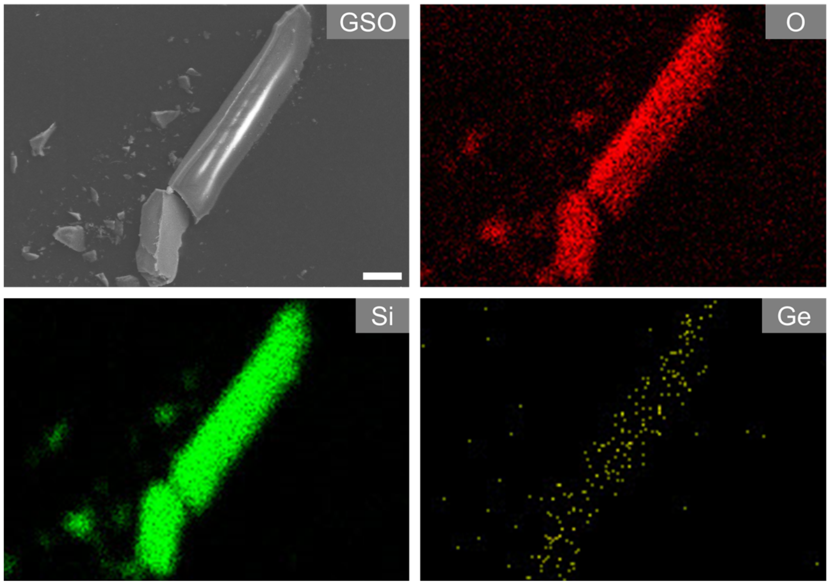

Supplementary Fig. 1 | SEM image of GSO powders and the corresponding EDX mapping of O, Si, and Ge elements. Scale bar = 10 μm**.**

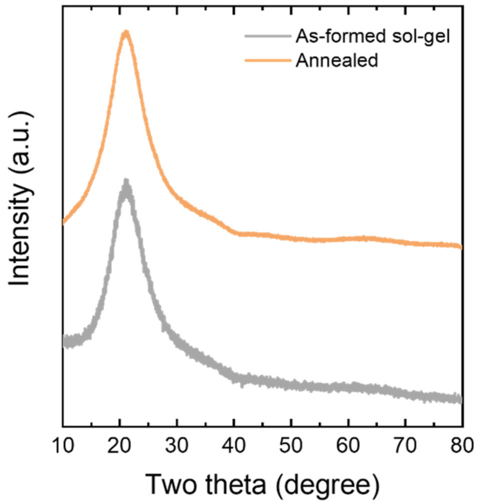

Supplementary Fig. 2 | PXRD patterns of GSO before and after thermal annealing. a.u. is the abbreviation of arbitrary units. The broad signals at $2\theta = 22°$ may originate from the amorphous silicon oxide matrix.

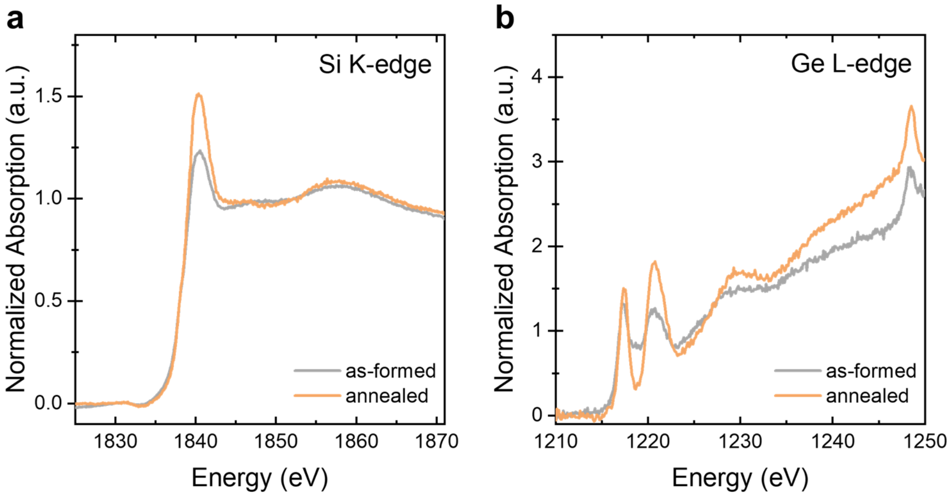

Supplementary Fig. 3 | X-ray absorption spectroscopy (XAS) of GSO. a-b, Si K-edge and Ge L-edge spectra of sample III before and after annealing, clearly showing the overall coordination environment of silicon and germanium atoms is not changed compared to those in the as-synthesized sol-gel precursor. a.u. is the abbreviation of arbitrary units.

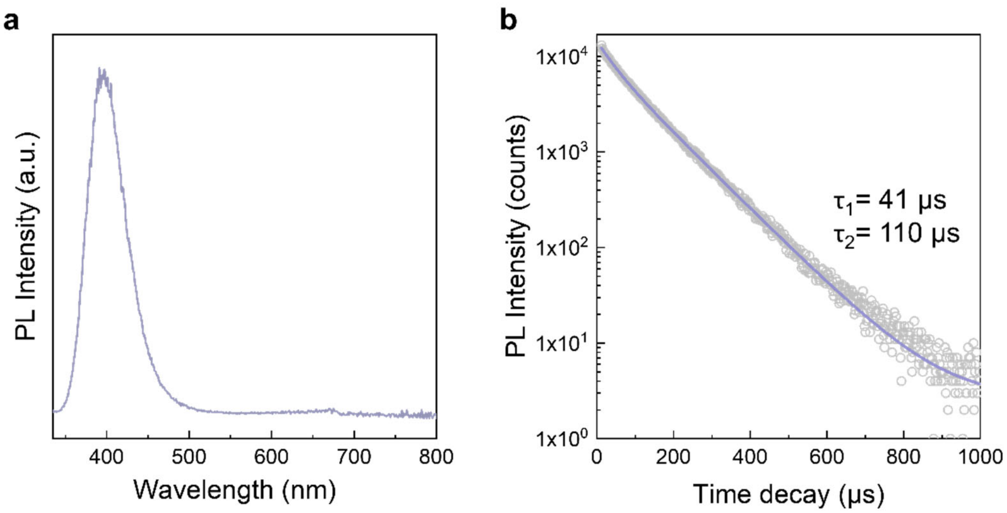

Supplementary Fig. 4 | PL properties of the GSO sample annealed at 800 °C. a, Steady-state PL spectra and **b,** PL lifetime decay of the emission at 395 nm (λ_{ex} = 250 nm). The photoexcited state lifetime decay values τ_1 and τ_2 are 41.2 μs and 110.4 μs, respectively. a.u. is the abbreviation of arbitrary units.

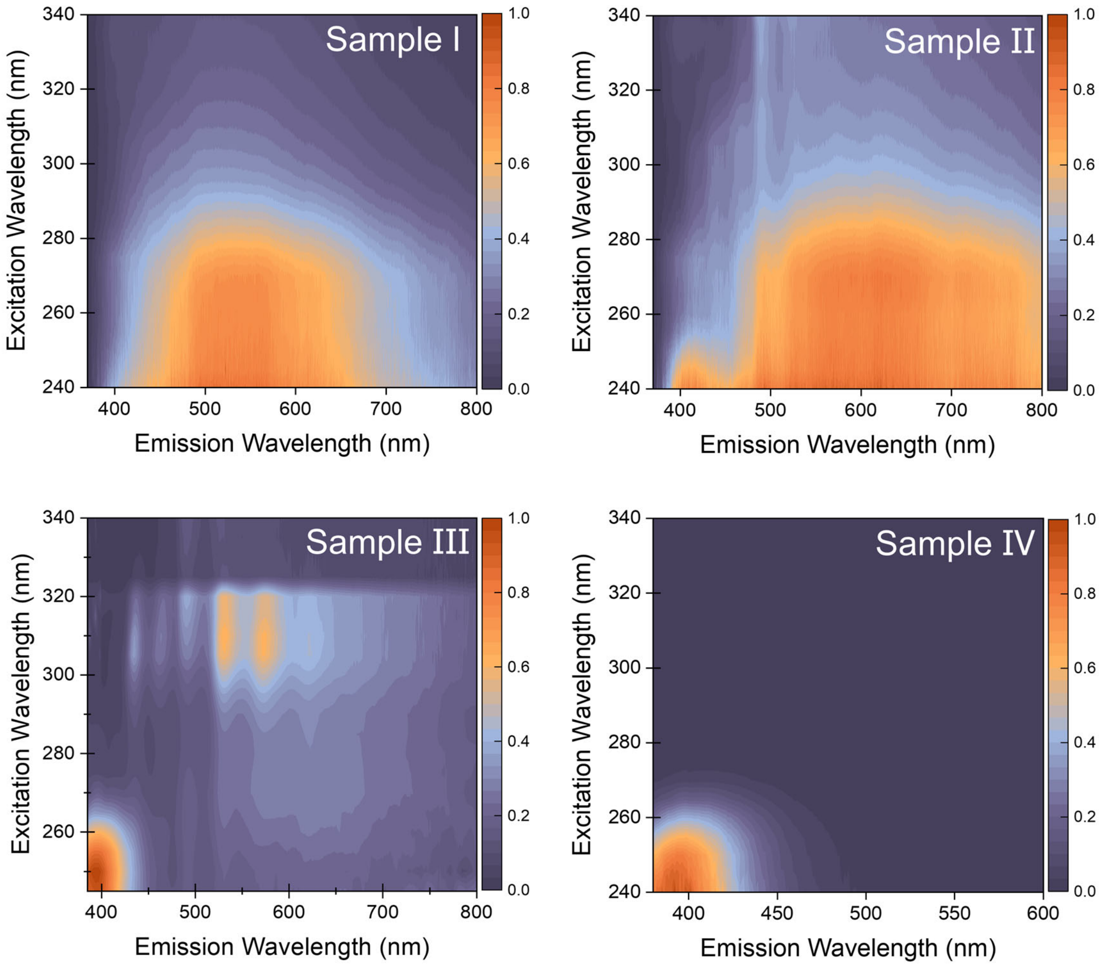

Supplementary Fig. 5 | EEM results of Samples I to IV. GSO samples are annealed at various temperatures (I: 550°C; II: 700°C; III: 800°C; IV: 900°C). The colour bar shows the normalized PL intensity.

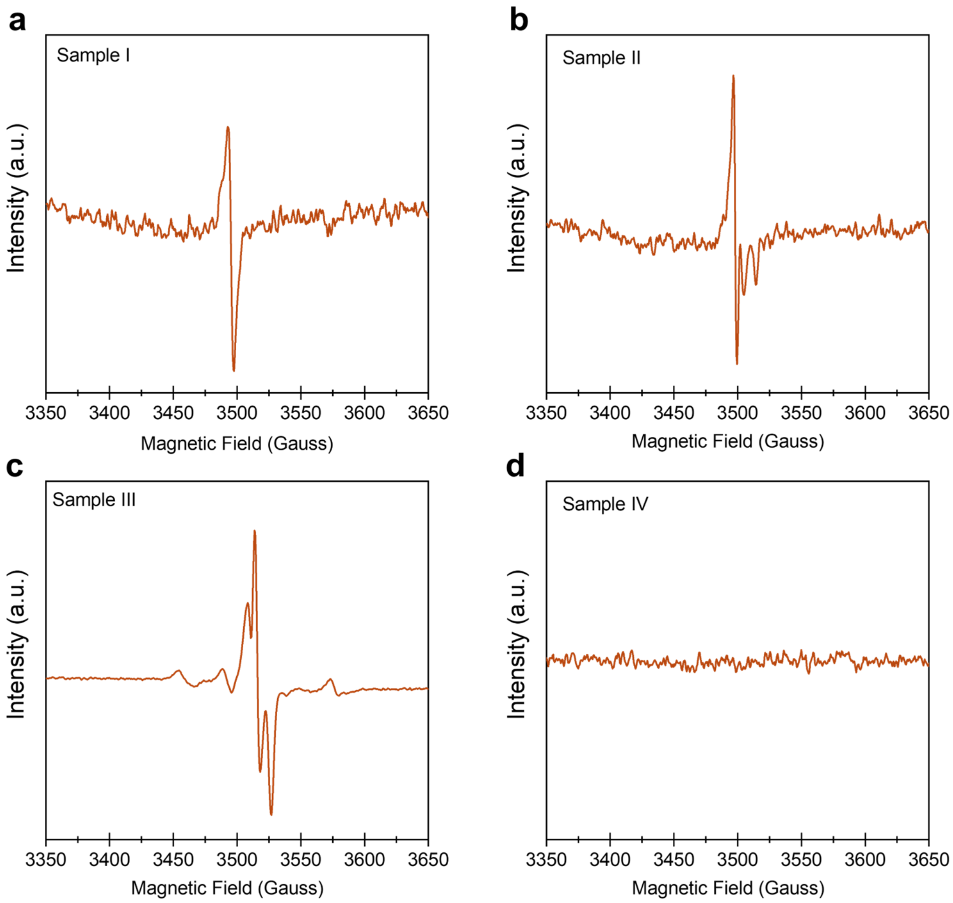

Supplementary Fig. 6 | EPR results of Samples I to IV. a-d, The EPR analysis was carried out on GSO samples annealed under different temperatures: (**a**) 550 ºC, (**b**) 700 ºC, (**c**) 800 ºC, and (**d**) 900 ºC. The results show the g-factor of the major paramagnetic defect decreases progressively going from Sample I to III – indicative of a tuned defect structure (Supplementary Table 2), whereas no EPR signal was detected for Sample IV: this agrees with a picture wherein diamagnetic GeODC(II) is substantially the only optically active defect created at this temperature. a.u. is the abbreviation of arbitrary units.

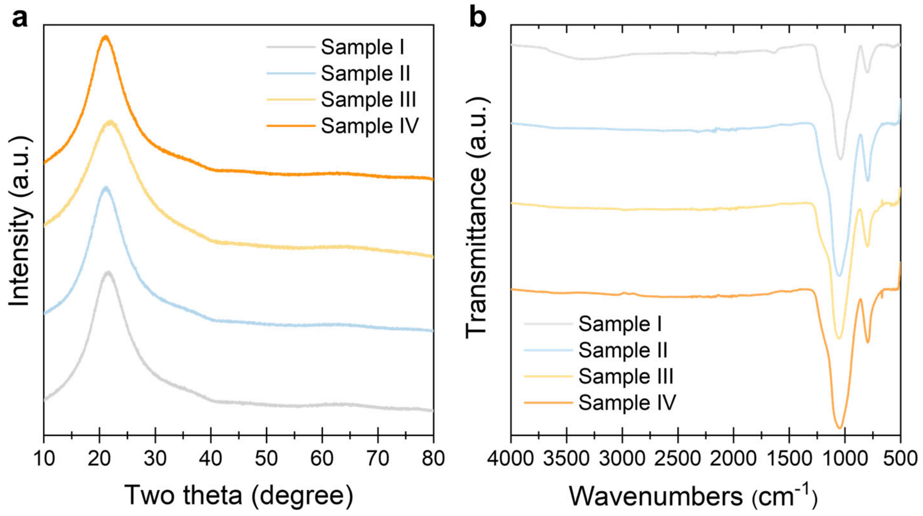

Supplementary Fig. 7 | Structural analysis of GSO samples. a, PXRD patterns and **b,** FT-IR spectra of Samples I to IV. Sample I-IV represents GSO samples annealed at different temperatures (I: 550°C; II: 700°C; III: 800°C; IV: 900°C). a.u. is the abbreviation of arbitrary units.

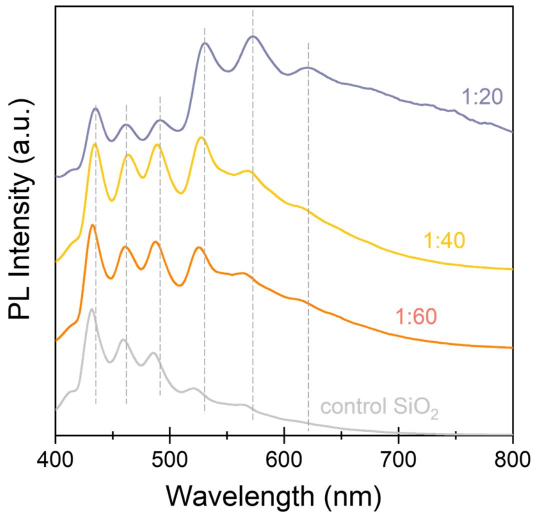

Supplementary Fig. 8 | PL spectra of GSO samples with different Ge:Si atomic ratios (excitation wavelength = 300 nm). Control SiO_2 and GSO samples with a Ge-doping concentration of 1:60, 1:40, and 1:20 were measured, respectively. a.u. is the abbreviation of arbitrary units.

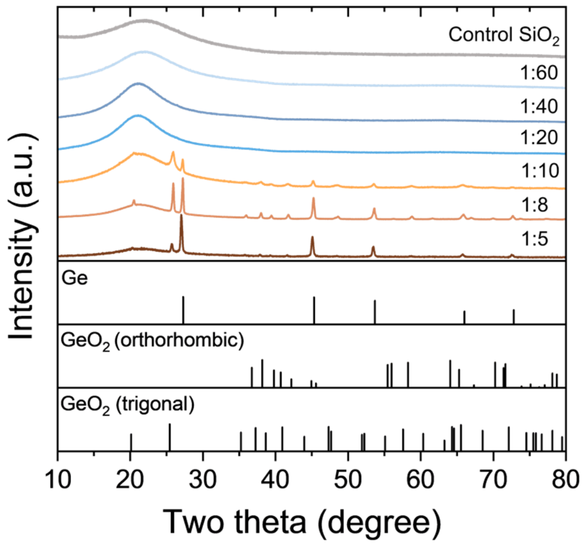

Supplementary Fig. 9 | XRD patterns of GSO with different Ge:Si atomic ratios. JCDPS Reference: Ge: #04-0545; GeO_2 (orthorhombic): #34-1089; GeO_2 (trigonal): #43-1016.

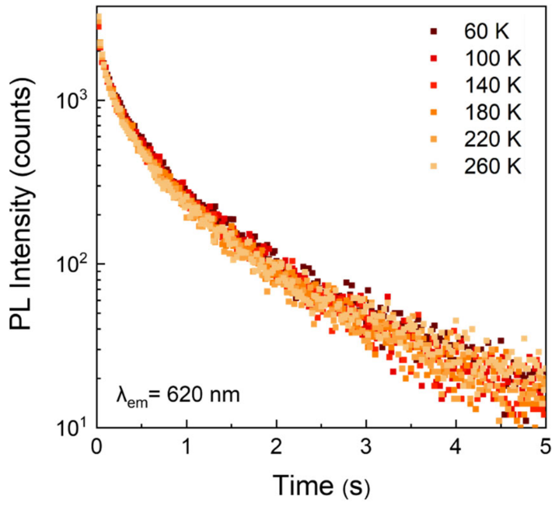

Supplementary Fig. 10 | Temperature-dependent PL lifetime decay of GSO. The PL decay curves were recorded for 620 nm PL emission with temperature in the range from 60 K to 260 K.

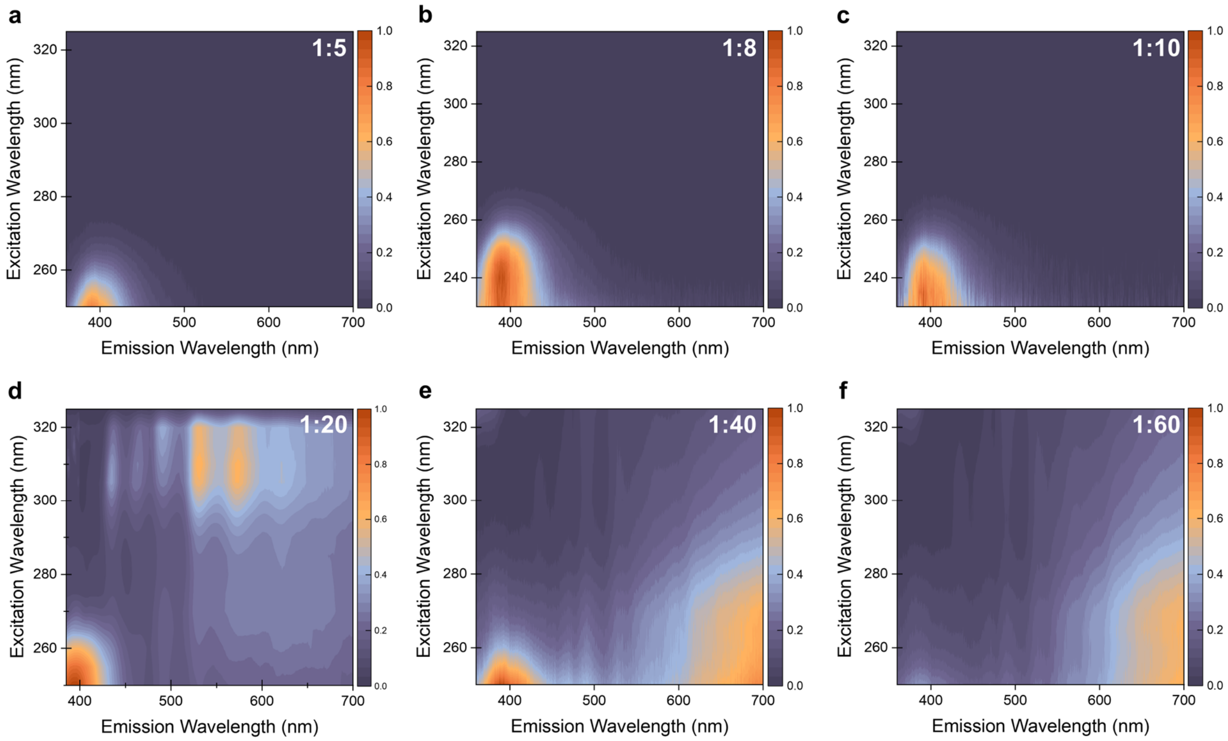

Supplementary Fig. 11 | a-f, The EEM spectra of GSO samples with a Ge doping concentration of (**a**) 1:5, (**b**) 1:8, (**c**) 1:10, (**d**) 1:20, (**e**) 1:40, and (**f**) 1:60. The colour bar shows the normalized PL intensity.

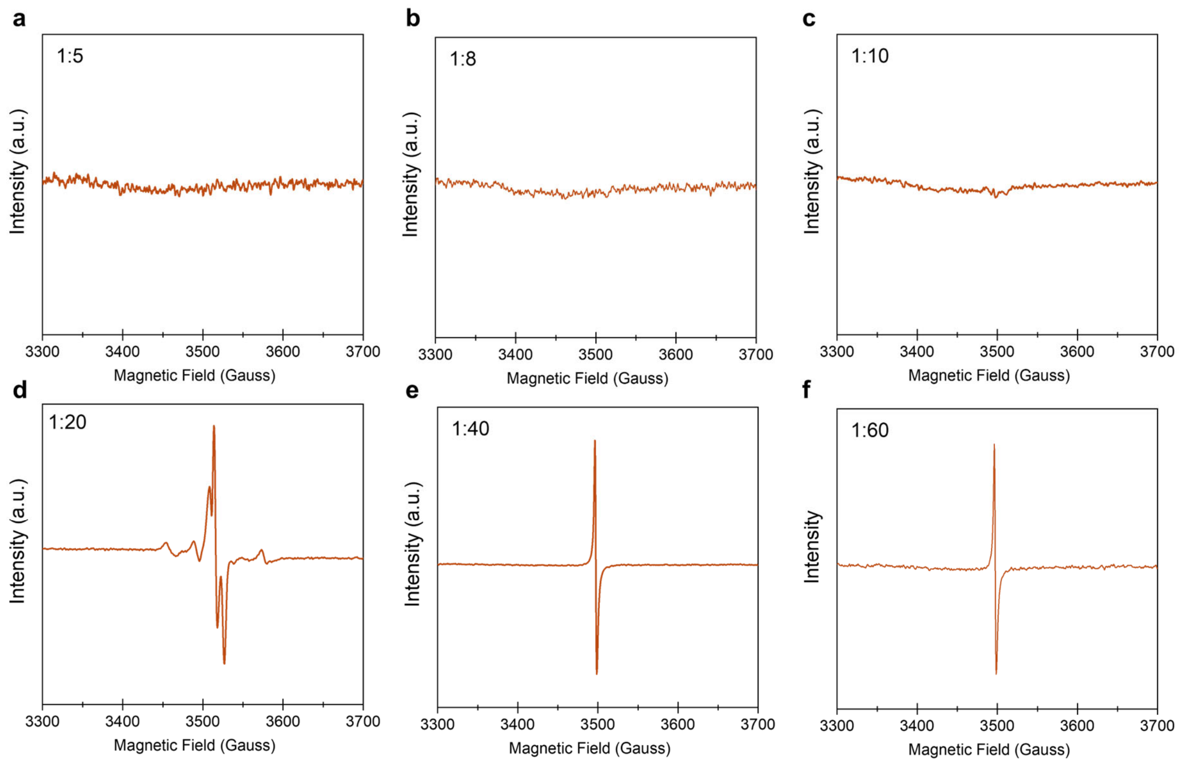

Supplementary Fig. 12 | EPR spectra of GSO samples prepared from the thermal annealing (800°C, 5 h) of the sol-gel precursors with different Ge:Si atomic ratios. a-f, The EPR analysis was carried out on GSO samples with different Ge doping concentrations: (**a**) 1:5, (**b**) 1:8, (**c**) 1:10, (**d**) 1:20, (**e**) 1:40, and (**f**) 1:60. a.u. is the abbreviation of arbitrary units. Only a Si-related defect was observed at low Ge doping concentrations (1:40 and 1:60), while an additional Ge-related defect was found at the Ge doping concentration of 1:20. For doping concentrations higher than 1:20, no EPR signal was detected, which we explain by noting the diamagnetic nature of the GeODC(II) defect. The fact that Ge- and Si-related paramagnetic defects coexist only at the doping level of 1:20, where the sensitization of Si LCs occurred, agrees with the picture of energy transfer from Ge defects to Si LCs.

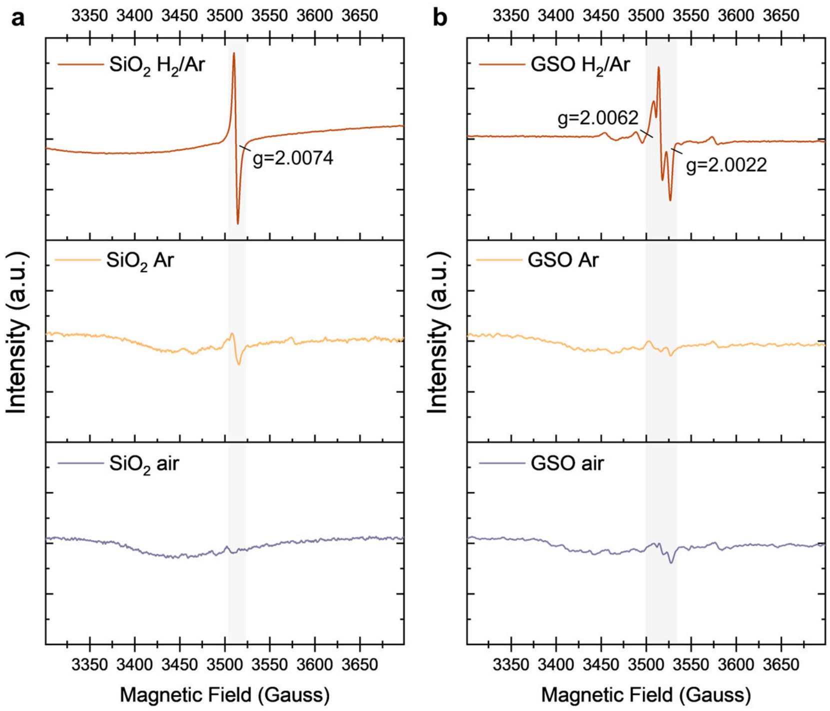

Supplementary Fig. 13 | EPR spectra of the control and GSO samples annealed under different atmospheres. a, EPR spectra of the control SiO_2 samples and **b,** GSO samples annealed under a slightly reducing atmosphere (5% H_2 + 95% Ar), Ar, or in air. a.u. is the abbreviation of arbitrary units. When we vary the annealing atmosphere during synthesis, we find that these defects are hydrogen-related defect centres.

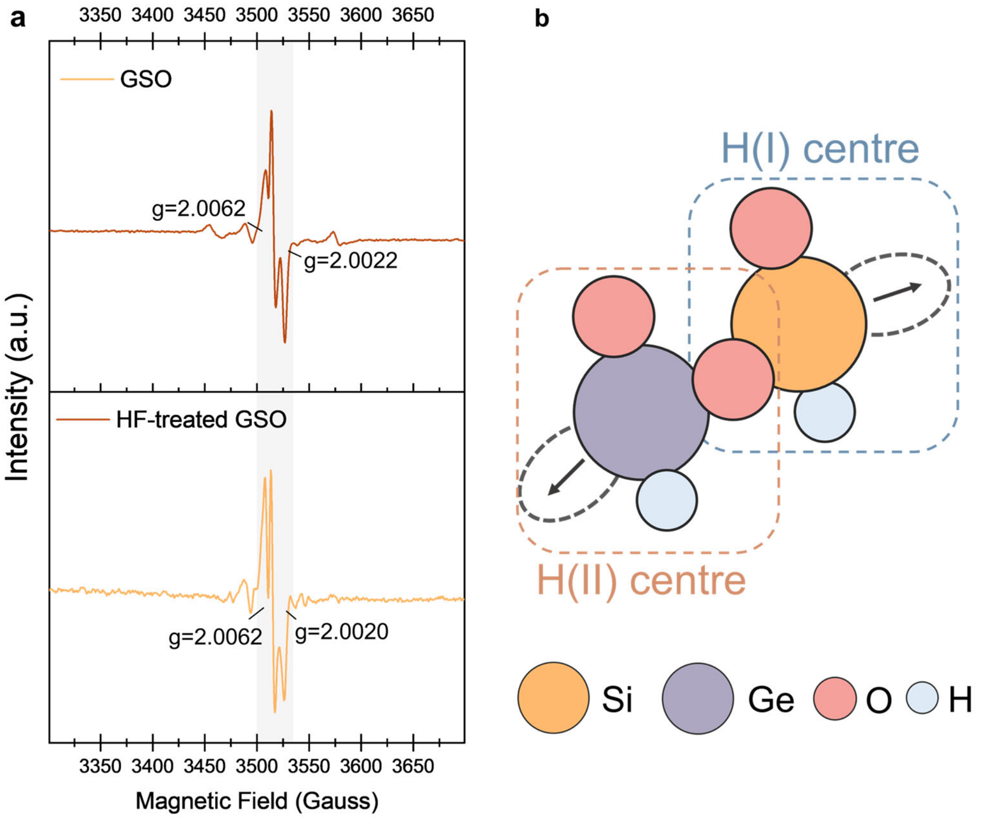

Supplementary Fig. 14 | EPR spectra of Sample III before and after hydrofluoric acid (HF) etching treatment. a, EPR spectra of Sample III before and after HF-etching. **b,** Models of the EPR-active H(I) and H(II) centres. a.u. is the abbreviation of arbitrary units. Two paramagnetic defects are identified at g = 2.0062 and 2.0022, respectively. The proportion of the defect at g = 2.0062 increased when we etched GSO using HF solution, indicating this is a Ge defect instead of a Si defect, which we assign to the H(II) centre.

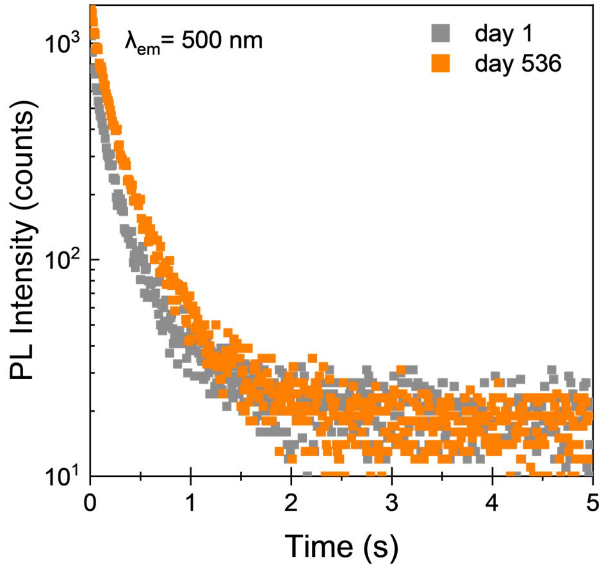

Supplementary Fig. 15 | Air-storage photophysical stability of GSO evaluated by repeated PL lifetime measurement after 536 days. Negligible PL change with a lifetime of 0.47 s was measured on day 536 compared to the lifetime of 0.42 s measured on the first day. The emission wavelength is 500 nm.

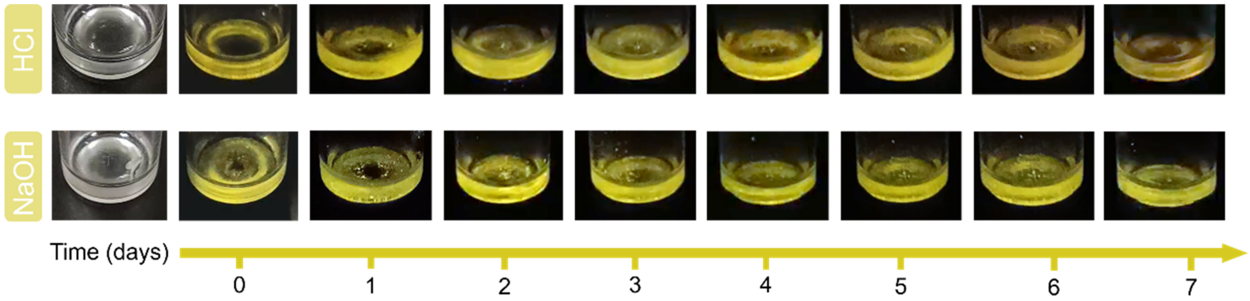

Supplementary Fig. 16 | Chemical stability tests of GSO. 10 mg GSO powders (Sample III) were immersed in HCl solution (1 mol/L) and NaOH solution (1 mol/L) at room temperature. Each photograph was shot at 1 s after switching off the excitation source (λ_{ex} = 365 nm). Negligible PL change for the after-glow emission after 7 days of tests.

Supplementary Table 1 | Performance summary of colour-tunable security phosphors.

**Material**	**Temp. range (K)**	**Emission tunability (nm)**	**Lifetime tunability (s)**	**Excitation wavelength responsive**	**Temp. responsive**	**Toxic element**	**Ref.**
TMOT	77 - 298	452 - 505	0.58 - 0.75	Yes	No	None	1
CBN /DOB	77 - 298	467 - 496	-	No	No	None	2
PAANa	77 - 443	441 - 568	0.46 - 0.89	Yes	No	None	3
CDs	77 - 298	525 - 630	0.1 - 0.8	Yes	No	None	4
PDNA	298	445 - 547	0.48 - 1.22	Yes	No	None	5
CDs @zeolite	77 - 298	430 - 466	-	No	Yes	None	6
CDs @SiO_2	77 - 480	418 - 507	-	Yes	Yes	None	7
F-CNDs	298	398 - 518	-	No	No	Sr, Pb	8
$FAPbBr_3$ {en}NCs	12 - 300	-	7×10^{-9} - 1×10^{-8}	No	Yes	Pb	9
$NaYF_4$:Yb/ Tm NCs	-	-	3×10^{-5} - 7×10^{-4}	No	No	Y, Tm, Yb	10
$Ca_{2-x}Eu_xSiO_4$	-	600 - 650	-	No	No	Eu	11
Tm^{3+}/Er^{3+}@ $NaYF_4$ NCs	-	980 - 1505	4×10^{-4} - 4×10^{-3}	Yes	No	Tm, Er	12
**GSO**	**60 - 500**	**395 - 525**	**10^{-4} - 0.58**	**Yes**	**Yes**	**None**	**This work**

Note: Phos. = phosphorescence, R.T. = room temperature, PVA = polyvinyl alcohol, CDs = carbon dots, DMFLTPD-d36 = N, N'-bis(3-methylphenyl)-N, N'-bis(phenyl)-9,9-dimethyl-fluorene, TMOT = 2,4,6-trimethoxy-1,3,5-triazine, CBN = cyanomethyl benzonitrile, DOB = 4-(2-(4-(diphenylamino) phenyl) -2-oxoethyl) benzonitrile, PAA = polyacrylic acid, F-CNDs = Folic acid-derived carbon nanodots, {en} = ethylenediammonium, NCs = nanocrystals

Supplementary Table 2 | *g*-factor from the major paramagnetic defect in GSO vs. annealing temperature.

Temp. (°C)	*g*-factor value
550	2.0078
700	2.0076
800	2.0022

Supplementary Table 3 | *g*-factor associated with the H(I) centre on GSO as a function of Ge: Si atomic ratio after thermal annealing at 800°C for 5 h under a slightly reducing atmosphere (5% H_2 + 95 Ar).

Ge:Si atomic ratio	*g*-factor value of H(I) on GSO
1:20	2.0022
1:40	2.0062
1:60	2.0070
Control SiO_2 sample	2.0074

Supplementary Note 1 | Electron paramagnetic resonance (EPR) studies of GSO and the control SiO_2 samples

EPR is a powerful tool to investigate the paramagnetic-active states of materials. Here we performed EPR studies on the origins of Si and Ge LCs in GSOs. The strong doublet EPR signals at $g = 2.0074$ were seen in the control SiO_2 sample prepared in the reducing atmosphere, indicating the presence of the H(I) centre related to SiODC(II) defect which has been considered to be the origin of the yellow PL[13,14] (Supplementary Fig. 13a and 14b). This is further supported by the fact that the signal is significantly reduced from the SiO_2 sample prepared in Ar or air (Supplementary Fig. 13a). As for GSOs, because the violet-emitting GeODC(II) state is EPR silent[14], we instead investigated the behaviour of the GeODC(II)-relavant H(II) centre (Supplementary Fig. 14b). Notable EPR features at $g = 2.0022$ assigned to GeODC(II) are found from the Sample III GSO annealed in H_2/Ar (Supplementary Fig. 6 and 13). The drastically reduced signal of GeODC(II) and the disappearance of long-live yellow PL in the GSOs annealed without H_2 further suggest that the H-related states are important to the efficient energy transfer between LCs in GSOs.

To further verify the possible relationship between the defect type and the Ge concentration in GSOs, we conducted EPR analysis upon GSO samples with different Ge:Si ratios (Supplementary Fig. 12) and compare the corresponding EEM sand PL results (Supplementary Fig. 8 and Fig. 11). Only strong intensity of the H(II) centre can be observed from the highly luminescent Sample III with long PL lifetime decay, suggesting the strong relationship between

the EPR-active H(II) centre and the H(I) centre. This is also supported by the previous result by Pacchioni et al[15]. We reason that the fine-tunning of the local chemical environment of Si and Ge atoms is the key to forming the H(II) centre in the SiO_2 network for efficient energy transfer between Ge and Si LCs.

Supplementary References

1. Gu, L. *et al.* Colour-tunable ultra-long organic phosphorescence of a single-component molecular crystal. *Nat. Photon.* **13**, 406 (2019).

2. Tapavicza, E. *et al.* Generating function approach to single vibronic level fluorescence spectra. *J. Phys. Chem. Lett.* **10**, 6003 (2019).

3. Cai, S. *et al.* Enabling long-lived organic room temperature phosphorescence in polymers by subunit interlocking. *Nat. Commun.* **10**, 4247 (2019).

4. Zhou, B. *et al.* Boosting wide-range tunable long-afterglow in 1D metal–organic halide micro/nanocrystals for space/time-resolved information photonics. *Adv. Mater.* **33**, 2007571 (2021).

5. Gu, L. *et al.* Color-tunable ultralong organic room temperature phosphorescence from a multicomponent copolymer. *Nat. Commun.* **11**, 944 (2020).

6. Liu, J. *et al.* Carbon dots in zeolites: A new class of thermally activated delayed fluorescence materials with ultralong lifetimes. *Sci. Adv.* **3**, e1603171 (2017).

7. Sun, Y. *et al.* Temperature-responsive conversion of thermally activated delayed fluorescence and room-temperature phosphorescence of carbon dots in silica. *J. Mater. Chem. C.* **8**, 5744 (2020).

8. Green, D. C. *et al.* Controlling the fluorescence and room-temperature phosphorescence behaviour of carbon nanodots with inorganic crystalline nanocomposites. *Nat. Commun.* **10**, 206 (2019).

9. Yakunin, S. *et al.* Radiative lifetime-encoded unicolour security tags using perovskite nanocrystals. *Nat. Commun.*, **12**, 981 (2021).

10. Lu, Y. *et al.* Tunable lifetime multiplexing using luminescent nanocrystals. *Nat. Photon.* **8**, 32 (2014).

11. Sato, Y. *et al.* Tailoring of Deep-red luminescence in Ca_2SiO_4:Eu^{2+}. *Angew. Chem. Int. Ed.* **53**, 7756 (2014).

12. Zhang, H, *et al.* Tm^{3+}-sensitized NIR-II fluorescent nanocrystals for In vivo information storage and decoding. *Angew. Chem. Int. Ed.* **58**, 10153 (2019).

13. Salh, R. *et al*. Defect related luminescence in silicon dioxide network: A review (2011).

14. Skuja, L. *et al.* Isoelectronic series of twofold coordinated Si, Ge, and Sn atoms in glassy SiO_2: a luminescence study. *J. Non-Cryst. Solids* **149**, 77 (1992).

15. Pacchioni, G. Ferrario R. Optical transitions and EPR properties of two-coordinated Si, Ge, Sn and related H(I), H(II), and H(III) centers in pure and doped silica from ab initio calculations. *Phys. Rev. B* **58**, 6090 (1998).
