# Supplementary material for: Germanium silicon oxide achieves multi-coloured ultra-long phosphorescence and delayed fluorescence at high temperature
Source: Nat Commun. 2022 Aug 1;13:4438. doi: 10.1038/s41467-022-32133-2 (PMC9343423; doi:10.1038/s41467-022-32133-2)
Supplement: Supplementary file 2 — Description of Additional Supplementary Files [file 41467_2022_32133_MOESM2_ESM.pdf]

Description of Additional Supplementary Files

File Name: Supplementary Data 1
Description: Yellow-coloured afterglow from germanium doped silica after switching off a 365 nm light illumination
